# Forest top canopy bacterial communities are influenced by elevation and host tree traits

**DOI:** 10.1186/s40793-024-00565-6

**Published:** 2024-04-05

**Authors:** Yiwei Duan, Andjin Siegenthaler, Andrew K. Skidmore, Anthony A. Chariton, Ivo Laros, Mélody Rousseau, G. Arjen De Groot

**Affiliations:** 1 Faculty of Geo-Information Science and Earth Observation (ITC), University of Twente, Drienerlolaan 5, PO Box 217, 7500 AE Enschede, The Netherlands; 2 School of Natural Sciences, Macquarie University, Sydney, NSW Australia; 3 Wageningen Environmental Research, Wageningen UR, P.O. Box 46, 6700 AA Wageningen, The Netherlands

**Keywords:** Phyllosphere, Microbiome, Biodiversity, *16S rRNA* gene, Temperate European forests

## Abstract

**Background:**

The phyllosphere microbiome is crucial for plant health and ecosystem functioning. While host species play a determining role in shaping the phyllosphere microbiome, host trees of the same species that are subjected to different environmental conditions can still exhibit large degrees of variation in their microbiome diversity and composition. Whether these intra-specific variations in phyllosphere microbiome diversity and composition can be observed over the broader expanse of forest landscapes remains unclear. In this study, we aim to assess the variation in the top canopy phyllosphere bacterial communities between and within host tree species in the temperate European forests, focusing on *Fagus sylvatica* (European beech) and *Picea abies* (Norway spruce).

**Results:**

We profiled the bacterial diversity, composition, driving factors, and discriminant taxa in the top canopy phyllosphere of 211 trees in two temperate forests, Veluwe National Parks, the Netherlands and Bavarian Forest National Park, Germany. We found the bacterial communities were primarily shaped by host species, and large variation existed within beech and spruce. While we showed that there was a core microbiome in all tree species examined, community composition varied with elevation, tree diameter at breast height, and leaf-specific traits (e.g., chlorophyll and P content). These driving factors of bacterial community composition also correlated with the relative abundance of specific bacterial families.

**Conclusions:**

While our results underscored the importance of host species, we demonstrated a substantial range of variation in phyllosphere bacterial diversity and composition within a host species. Drivers of these variations have implications at both the individual host tree level, where the bacterial communities differed based on tree traits, and at the broader forest landscape level, where drivers like certain highly plastic leaf traits can potentially link forest canopy bacterial community variations to forest ecosystem processes. We eventually showed close associations between forest canopy phyllosphere bacterial communities and host trees exist, and the consistent patterns emerging from these associations are critical for host plant functioning.

**Supplementary Information:**

The online version contains supplementary material available at 10.1186/s40793-024-00565-6.

## Background

The phyllosphere consists of the aerial parts of plants, primarily photosynthetic leaves, which provides a unique microbial habitat [1]. Phyllosphere bacterial communities encompass bacteria associated with plant leaves both epiphytically and endophytically [2, 3] and play a vital role in host plant function and ecosystem processes [4–7]. Despite its important, the phyllosphere microbiome remains understudied compared the rhizosphere microbiome [8]. The plant microbiome participates in host-environment interactions by being involved in plant nutrition and stress responses [6], forming a holobiome with its host [9]. These interactions manifest in various forms, including plant pathogen infections [10], plant-microbes symbioses [11], and the recruitment of both disease-fighting and growth-promoting bacteria by plants [12]. In a forest, the interactions among a diverse array of holobionts, each varying in longevity, size, and reproductive success, contribute to the dynamic nature of forest ecosystems, influencing the overall biogeochemical cycle [13]. Therefore, the variation in the microbes inhabiting the phyllosphere could be a manifestation of changes in plant communities, subsequently influencing the ecological processes of the forest ecosystem. Under the multitude of environmental stressors temperate European forests are facing today (e.g., droughts [14], pests [15], aerial nitrogen deposition [16]), it is urgent to formulate a baseline understanding of this important ecological niche.

Within the plant phyllosphere, a few themes consistently emerge: (1) bacteria are believed to be the most predominant microbes in terms of density and diversity [17], (2) often core communities exist (here defined as taxa present in at least 99% of samples, exact threshold can vary between studies [2, 18]), and (3) host species is the primary driver of the phyllosphere bacterial communities [19]. Although multiple larger scale studies on forest phyllosphere from various regions, including temperate forests in Quebec [2], and tropical forest in Panama [20], already exist, comprehensive studies examining the phyllosphere microbiome in temperate European forests across tree species, forest stands, and regions are infrequent [21]. Assessing the phyllosphere also in these forests is of relevance, given the documented influence of geography on phyllosphere microbiome [22–24].

Although we know the phyllosphere microbiome mainly differentiates between host tree species [19], it is also important to investigate the variation within the confines of a single tree species to gain a more nuanced understanding of plant microbiome assembly and the extent of intra-specific variabilities. This may be of specific relevance in temperate European forest which are, compared to tropical forests, dominated by only a few tree species [25]. While studies show that certain host traits such as tree height [18], adjacent plant species richness [26], geographic location [24], sampling season [27], climatic conditions [28], and various leaf traits (e.g., carbon and nitrogen content, chlorophyll content, leaf mass per area) [20] influence the leaf microbial communities, comprehensive studies assessing these driving factors and their large-scale ecological implications are rare (but see: [29]).

An important aspect of forests compared to other ecosystems, such as agricultural or grass landscapes, is the large variation in vertical structure, with many trees growing to heights above 30 m. Height of the trees is of particular relevance since canopy position could play a pivotal role in microbial community assembly [30]. Phyllosphere communities are generally characterized using samples collected from easy-to-reach heights [31, 32]. In a study where the forest top canopy leaves were collected by a crane, the top canopy phyllosphere constituted a unique microbiome that was significantly different from the middle and bottom canopies, suggesting canopy position had a stronger influence on the microbial communities that host species [30]. This is not surprising given the top canopy is influenced by a different set of environmental factors like UV radiation, desiccation, rain events, and temperature oscillation, besides being an oligotrophic environment [19], qualifying it as an extreme environment. In comparison, conditions are milder in the middle and bottom canopies where soil can also be a more influential factor, as it has been shown to act as a reservoir for phyllosphere bacteria [33, 34].

Therefore, in this study, we aim to investigate the variation in the top canopy phyllosphere bacterial communities’ diversity and community composition between and within host tree species in two temperate European forests, looking at the combination of epiphytic and endophytic bacteria. We will address the following research questions: How prominent is the role of tree species in the diversity and composition of bacterial communities in the top canopy phyllosphere? And within one host tree species, what is the degree of variation in its phyllosphere bacterial community diversity and composition? Can the intra-specific variation be explained by elevation and host traits (including leaf-specific traits)? Finally, do the identified explanatory variables for intra-specific variation also correlate with discriminant bacterial taxa? To answer these questions, we focus specifically on the phyllopshere of six common tree species in temperate European forests: *Fagus sylvatica* (European beech), *Picea abies* (Norway spruce), *Quercus robur* (European oak), *Pinus sylvestris* (Scots pine), *Abies alba* (silver fir), and *Betula pendula* (European birch).

## Methods

### Study design and sample collection

The Veluwe forest area in the Netherlands and the Bavarian Forest National Park in Germany are two distinct forested areas that are representative of the temperate European forest landscape. The Veluwe forest area consists of different parks covering 900 km^2^ of forests, heathlands, and sand dunes [35]. In this study, two of these parks were sampled: Het Nationale Park De Hoge Veluwe and Nationaal Park Veluwezoom, referred here together as Veluwe National Park. Among its prominent tree species are beech, Scots pine, and oak. Meanwhile, the Bavarian Forest National Park, part of the Bohemian Forests ecosystem, spans about 2,000 km^2^ in Central Europe [36], and hosts intensive biodiversity and forestry research [37]. At lower elevations, beech and spruce form the dominant tree species in Bavarian Forest National Park [38].

**Fig. 1:**
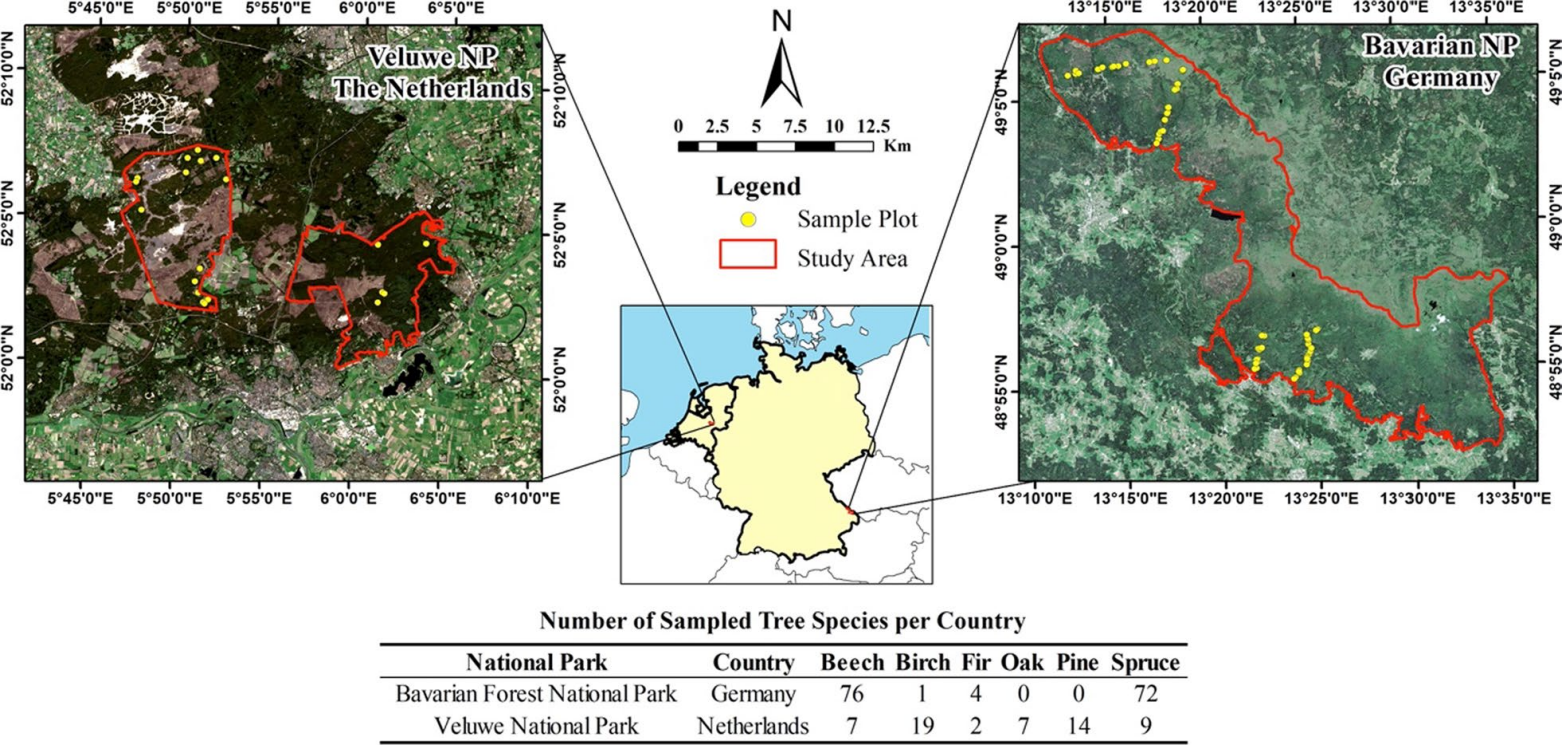
Study sites and sampling design. Within the Veluwe National Park, samples were collected in two specific areas: Het Nationaal Park De Hoge Veluwe (left) and Het Nationaal Park Veluwezoom (right)

We sampled 211 trees from six tree species in Bavarian Forest National Park and Veluwe National Park (Fig. 1) during May–July 2020, when broadleaves were fully mature and before senescence. A total of 45 and 19 plots were sampled, respectively, in Bavarian Forest National Park and Veluwe. Each plot was 30 m × 30 m. Within each plot, two to five trees representative of the plot in terms of tree species composition, tree height, and canopy size were selected for sampling, with an average of three trees sampled per plot. Sampling followed a stratified random design over tree species, elevation gradient, and forest stand age. With spruce and beech being the two dominant tree species in Bavarian Forest National Park, our stratified sampling by species resulted in a larger sample size for these two species (Fig. 1). This allowed for a representative sample, a wide inter-specific variation, and high resolution of intra-specific variation within the two most common tree species. For each tree, rope and either a slingshot (Tree runner BigShot) or a cross-bow were used to collect leaves from the top, sun-lit canopies [39]. For each tree, an average of eight grams of leaf material was collected to use for phyllosphere bacterial communities metabarcoding and biochemical analyses. Leaf samples were analyzed for a range of biochemical traits, including chlorophyll content, leaf nitrogen, carbon, phosphorus, calcium, potassium, magnesium, zinc, iron, and manganese. Additionally, leaf physiological traits including leaf mass per area (LMA) and water content were assessed. Collected geographical data and host tree traits included tree diameter at breast height (DBH), geographical coordinates, and elevation. Ten leaves or needle branches that were representative of the canopy were picked from the sample pool to represent the sampled tree. Single use sterile bags and gloves were used to prevent cross-contamination. Contamination of DNA samples was avoided during sampling by ensuring they do not come into contact with soil or ground vegetation. Majority of the samples were kept in a cool box and transferred to a – 20 °C freezer by the end of the day, except 39 Veluwe samples that were snap-frozen in liquid nitrogen (due to logistical considerations) before being transferred to the freezer. No significant differences in bacterial communities were found between the two field storage methods, as indicated by the analysis of the same samples divided to be stored using two different methods (Additional file 1: Table S1). Therefore, samples were treated indiscriminately during downstream processing and analyses, irrespective of the field storage method.

### Field measurements

Fresh weight of collected leaf samples was measured immediately after sampling, and LMA was determined the same day using an ADC AM350 leaf area meter. Tree DBH was measured using a DBH meter. Tree canopy (crown) diameter was measured using a range finder. Forest stand type was also documented. Plot center location was determined using the Differential Global Positioning System (DGPS) (Leica GPS 1200) with an accuracy of 0.24 m after removing outliers. Based on plot coordinates, elevation was extracted from digital elevation model (DEM) using ArcMap 10.8.2. The DEM was generated from airborne LiDAR point clouds with 1 m resolution [40, 41].

### Leaf biochemistry

Leaf material used for chlorophyll measurement was protected from light using aluminum foil upon gathering and measured using VWR UV–VIS Spectrophotometer by digesting 0.05 g of frozen leaf material in MgCO_3_ buffered acetone [42]. The remaining leaves were oven dried for 48h, milled using a household coffee grinder, and used for leaf carbon, nitrogen, and micro-nutrients measurements. Carbon and nitrogen content were measured using Perkin Elmer 2400 CHN/O Series II System per manufacturer’s instructions. Leaf micro-nutrients concentration for P, K, Ca, Mg, Mn, Fe, and Zn were obtained using an inductively Coupled Plasma—Optical Emission Spectrometry (Perkin Elmer 8300DV ICP/OES) following open digestion of 200 mg of dried leaf material in a 6 ml nitric acid and hydrogen peroxide solution (1:1 volume). Element recovery rates were determined by digesting and measuring a certified reference material (Apple Leaves NIST® SRM® 1515). Blanks were used during digestion and measurements as negative controls.

### DNA extraction and sequencing

For each tree, 0.1 g of leaf material was pooled from a composite of ten leaves / needle branches. A sterile paper hole puncher (0.6 cm Ø) was used to obtain leaf disks from broadleaf samples. Without surface sterilization in order to preserve both epiphytic bacteria and endophytic bacteria, sample was homogenized using Benchmark Beadbug™ Mini Homogenizer (D1030). Total genomic DNA was extracted from the leaf samples using the Qiagen Dneasy Plant Pro Kit and the Qiagen Qiacube Connect, following kit manual and manufacturer’s protocol. Samples were processed in batches containing 22 samples each. DNA concentration of each sample was quantified using the Quant-iT PicoGreen dsDNA Assay Kit and Biotek Synergy HTX Multi Mode Reader. DNA extracts were then normalized to 5 ng/ml, with the exception of samples with a lower concentration. Bacterial *16S rRNA* gene V3-V4 region was targeted for amplification using the 515F/806R primer sets [43, 44]. Prior to PCR, negative extraction controls were combined. To mitigate amplification of plant host DNA, peptide nucleic acid (PNA) clamps [45, 46] were used. Their efficacy was determined through a comparison using 12 sample pairs (four tree species represented by three pairs each) amplified with, and without PNA clamps. Amplification protocols and polymerase chain reaction (PCR) reagents mixture are shown in Additional file 1: Tables S2 and S3. To mitigate and control for DNA contamination [47], we implemented (1) a negative extraction control with every batch of sample DNA extraction, (2) two negative PCR controls per 96-well plate, and (3) three spike-in positive controls (Additional file 1: Table S2) per 96-well plate to control for cross-contamination and tag-switching. Each control type was pooled and sequenced separately. Library preparation and amplicon sequencing (Illumina NovaSeq 6000 SP paired-end 250 bp) were performed by Genome Quebec (Montreal, Canada). During library preparation, multiplexing using the Fluidigm Access Array System (Fluidigm, South San Francisco, CA) was adopted with CS1 (forward primer) and CS2 (reverse primer). An indexing PCR (15 cycles) was used to attach the indexes and i5/i7 Illumina adapter sequences to the amplicons.

### Bioinformatic processing

QIIME2 pipeline [48] was used for bioinformatic analysis (Additional file 1: Table S4). The specific trimming positions of reads were selected after visual inspection of the sequence quality score plots. The maximum expected error rate of 2 is the default value of DADA2 [49]. Chimeras were removed (11% of reads). Reads unassigned at the kingdom level were also removed. Post-clustering curation was done using LULU [50], after which taxonomy was assigned using the SILVA database (version SSU 138) [51]. Low frequency noise (ASVs ≤ 5 reads) was removed [52]. Data was then rarefied at 10,312 reads per sample (based on minimum reads after removal of all samples < 10,312 reads) with averaging 99 iterations. Rarefaction was performed using the ‘rrarefy’ function from the vegan package (version 2.6-4) [53] in R (version 4.2.3) [54].

### Statistical analyses

Bacterial alpha diversity was calculated using the ‘estimate_richness’ function from the phyloseq package [55]. The R package ‘ggplot2’ [56] and ‘microeco’ [57] were used for visualization. All statistical tests were performed at a significance level of 0.05. Principal component analysis (PCA) was used to visualize variation in elevation and host traits between the different tree species. Bacterial alpha diversity (Shannon index and richness) and community structure variation were examined first between host tree species (inter-specifically) and then within host tree species (intra-specifically). Inter-specific comparative analyses were performed separately for Veluwe and Bavaria samples to minimize potential regional effects. Following our sampling design, within Veluwe forests, the comparative analysis involved five tree species: beech, spruce, pine, oak, and birch. In Bavaria, the comparison focused on beech and spruce. For Veluwe samples, inter-specific alpha diversity variation was compared using a Kruskal–Wallis test, after examining the normality of residuals and homoscedasticity. This was followed by pairwise Wilcoxon Rank Sum tests with Benjamini–Hochberg correction for multiple comparison. For Bavaria samples, this was compared using a Wilcoxon Rank Sum test. Inter-specific variation in community structure was analyzed using permutational multivariate analysis of variance (PERMANOVA [58]) on Bray–Curtis dissimilarities of Hellinger-transformed reads utilizing the adonis2 function in package vegan. Hellinger transformation has been shown to yield lower model residuals during variation partitioning compared to non-transformed relative abundance [59, 60]. Then, intra-specific variation in diversity and community structure was assessed using a subset of samples, comprising beech and spruce samples from Bavarian National Park (n = 72 for spruce and n = 76 for beech), again to minimize regional effects between Veluwe and Bavaria. We first tested whether the intra-specific variation can be attributed to distance-decay using Mantel tests (999 permutations). Haversine distance, a more accurate measure than Euclidean distance when points are geographically distant, was used to calculate the distance between two sample points [61]. In Mantel tests, Pearson correlations between Bray–Curtis dissimilarities (based on Hellinger-transformed reads) and Haversine distance matrixes were determined [62, 63]. Following this, to identify the explanatory variables for intra-specific alpha diversity variation, linear regression with stepwise model selection based on Akaike Information Criterion (AIC) was used to select, and further, quantify the contribution of selected explanatory variables. To determine the explanatory variables of intra-specific variation in community structure, distance-based redundancy analysis (db-RDA) using Bray–Curtis distance of Hellinger-transformed reads was used to select the model with the lowest adjusted R^2^ (ordistep function). The contribution of the variables in the selected model was tested using PERMANOVA on Bray–Curtis dissimilarities of Hellinger-transformed reads between sample groups. To identify discriminant bacterial taxa, differential abundance analysis was performed at the bacterial family level on centered log ratio transformed read counts data using the ‘ALDEX2’ package [64]. We examined: (1) discriminant taxa between different tree species (aldex.ttest), with a minimal occurrence threshold of 10% of samples [65] and (2) discriminant taxa of elevation and host traits significant for intra-specific variation (aldex.corr) with a minimal occurrence threshold of 30% of samples.

## Results

### Sampling and DNA sequencing

Across the 211 leaf samples remaining after bioinformatic processing, quality filtering and rarefaction, 8972 ASVs and 2,175,905 bacterial reads were recovered (Additional file 1: Table S4), with an average of 365 ± 172 ASVs (mean ± std) and 10,312 ± 7 reads (mean ± std) per sampled tree. Plant reads (chloroplast and mitochondria) percentage ranged from 13.2% to 99.8% per sample (mean and std: 64.1 ± 21.6%). Synthetic spike-in sequences amplified in the positive control samples and were not detected in any other samples. Based on a comparison with duplicate amplicons without PNA clamps, using PNA clamps reduced 20% (from 91 to 71% (n = 12)) of plant reads (Wilcoxon signed rank paired test, V = 78, p < 0.01). However, being an “universal” plant DNA blocking agent, its efficacy in blocking plant DNA amplification varied between host tree species (Additional file 1: Table S5).

### Leaf biochemical traits

**Fig. 2:**
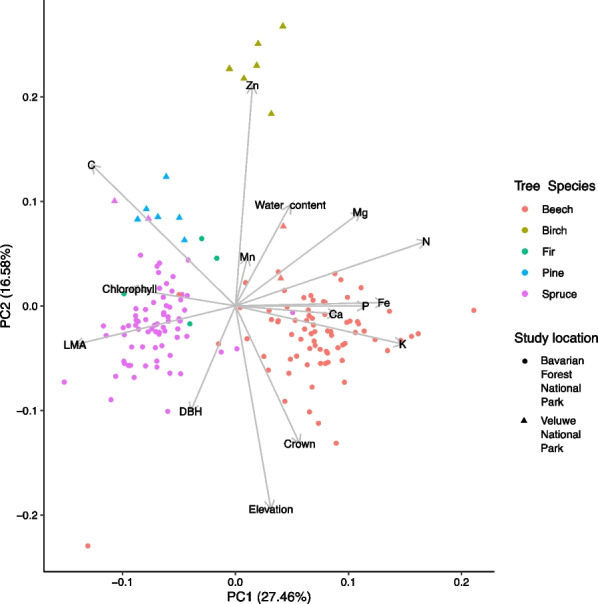
Differences in elevation and host tree traits among the studied tree species in Bavarian Forest National Park and Veluwe National Park as shown by principal component analysis. DBH: tree diameter at breast height, LMA: leaf mass per area

Examining the differences in elevation and host tree traits among the sampled tree species using PCA, it was evident these variables varied between and within tree species (Fig. 2 and Additional file 1: Table S6). The first principal component was associated with variables related to the ‘Leaf Economic Spectrum’ (e.g., chlorophyll, LMA and (micro) nutrients), a concept that measures plants’ resource investment to balance foliar growth with foliar longevity [66], and differentiated mainly trees from different tree functional types (coniferous vs. deciduous species). The second principal component was more associated with tree physiology (e.g., tree diameter at breast height) and differentiated samples within tree functional type (e.g., birch versus beech and pine versus spruce) as well as within tree species (especially within beech and spruce).

### Phyllosphere bacterial diversity among host tree species

**Fig. 3:**
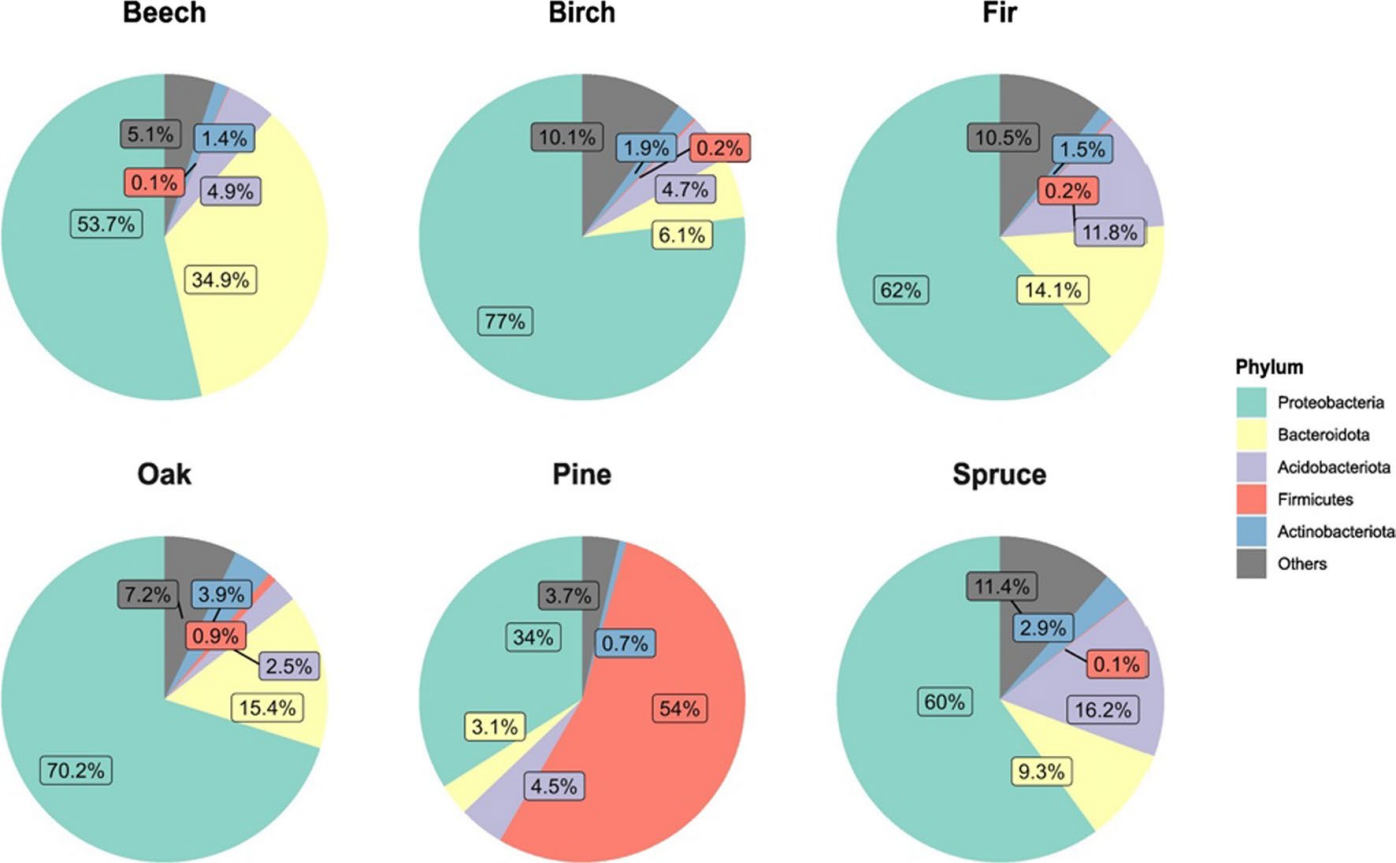
Top five phyla in the bacterial communities of temperate forest phyllosphere. Percentages represent relative read abundance

A total of 27 phyla, 65 classes, 162 orders, 277 families, and 525 genera of bacteria were identified, with most bacterial reads belonging to the phyla *Proteobacteria* (59.3% of all reads), *Bacteroidota* (19.5% of all reads), and *Acidobacteriota* (9.5% of all reads; Fig. 3). We found Scots pine harbored a Firmicutes-dominated (mean 54% in relative abundance, compared to < 1% in other species) leaf bacterial community (Fig. 3). *Proteobacteria* and *Bacteroidota* dominated beech, while Norway spruce and silver fir had the highest relative abundance of *Acidobacteriota*. The observed species-specific patterns are largely similar between Bavaria and Veluwe, with minor variations in specific percentage values (Additional file 1: Figure S1). Four ASVs (Additional file 1: Table S7) from the family *Beijerinckiaceae* and *Acetobacteraceae* were identified as the core taxa of temperate forest phyllosphere (present in > 99% of the field samples), with a detection threshold of 0.5% read abundance. These four core taxa combined represented 0.04% of the total ASVs, but 17.09% of total reads. ASV accumulation curves (Additional file 1: Figure S2) showed differences in total observed ASV richness between tree species. The curves, however, did not approach an asymptote, indicating the spatial heterogeneity of samples.

### Between host-species differences

**Fig. 4:**
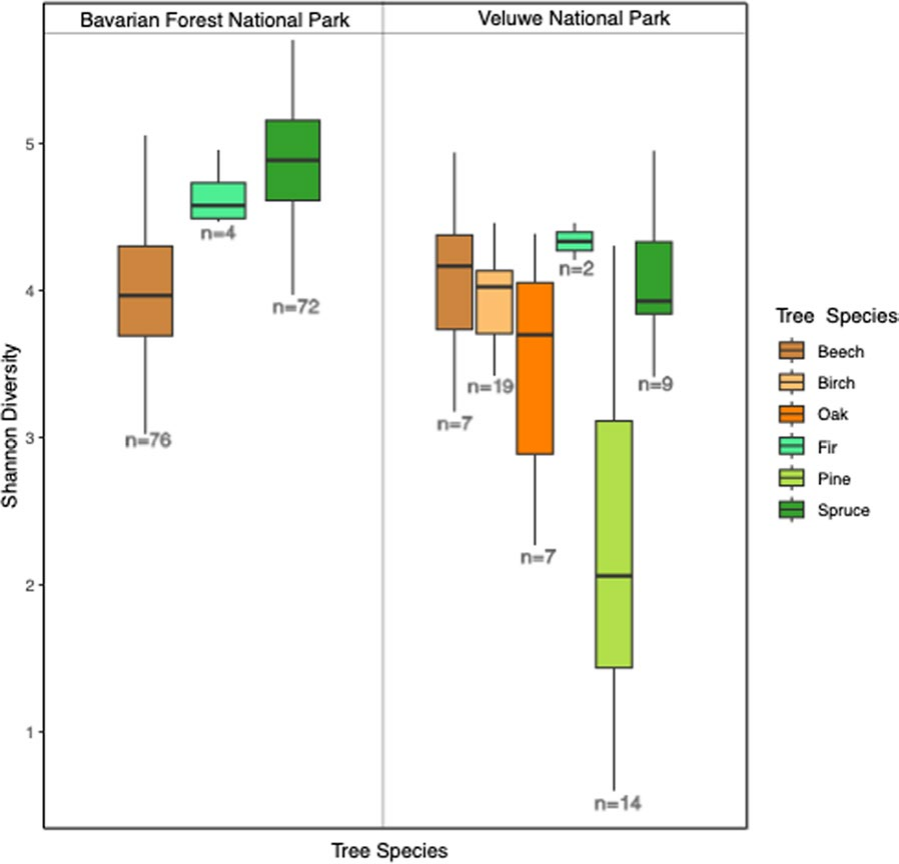
Boxplot showing the alpha diversity variation in phyllosphere bacterial communities (Shannon Index) across European temperate forest coniferous and deciduous tree species, rarefied to 10,312 reads/sample

**Fig. 5:**
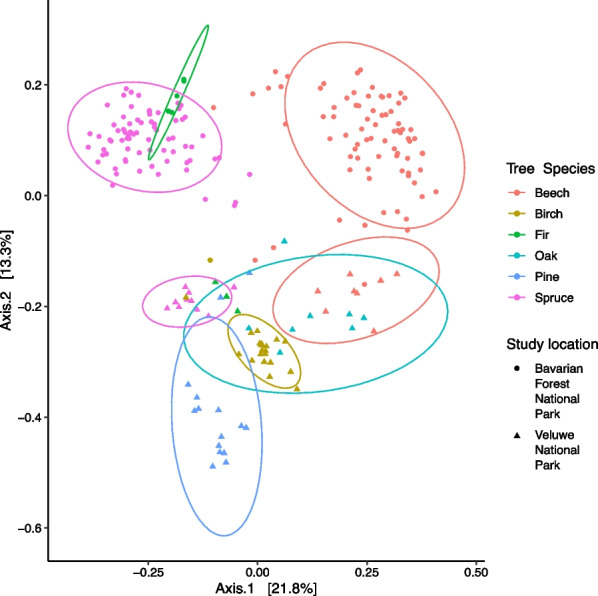
Variation in phyllosphere bacterial community composition (Bray–Curtis distance) in Bavarian Forest National Park and Veluwe National Park

There was significant variation (Additional file 1: Table S8) in phyllosphere bacterial alpha diversity (Shannon index and richness) among host tree species, both in Veluwe and Bavaria (Table 1 and Additional file 1: Table S8). In Veluwe: (1) Scots pine exhibited the lowest bacterial Shannon diversity and bacterial ASV richness (Fig. 4 and Additional file 1: Table S9); (2) coniferous species showed more variation in Shannon index compared to deciduous species (Fig. 4). In Bavaria, spruce had higher Shannon index and bacterial ASV richness compared to beech (Additional file 1: Tables S8 and S9). Similarly, there were also differences in bacterial community composition among the tree species that could be partially explained by host species (Fig. 5 and Table 2). All pairwise comparisons of bacterial community composition among tree species in Veluwe were also significantly different (Additional file 1: Table S10). Comparing the phyllosphere bacterial communities between beech and spruce from Bavaria using differential abundance analysis, we have identified two bacterial families to be differentially abundant in spruce, and five families to be differentially abundant in beech (Table 3).

**Table 1:** Inter-host species variabilities in bacterial Shannon diversity and the correlation between bacterial Shannon diversity and elevation, tree traits, based on step-wise linear regression. Samples have been rarefied to 10,312 reads/sample

Interspecific Veluwe	Coefficient	Std. Error	t	p-value	Adj. R^2^	F	p-value
Tree species birch	4.07	0.30	13.50	< 0.01	0.42	F(4, 51) = 10.88	< 0.01
Tree species oak	− 0.17	0.35	− 0.47	0.64			
Tree species pine	− 0.61	0.43	− 1.43	0.16			
Tree species spruce	− 1.76	0.37	− 4.77	< 0.01			
**Interspecific** **Bavaria**	**Coefficient**	**Std. Error**	**t**	**p-value**	**Adj. R**^**2**^	**F**	**p-value**
Tree species spruce	0.92	0.08	11.11	< 0.01	0.45	F(1, 146) = 123.3	< 0.01
**Spruce** **Intra-specific**	**Coefficient**	**Std. Error**	**t**	**p-value**	**Adj. R**^**2**^	**F**	**p-value**
Mg	− 0.0005	0.0001	− 3.8	< 0.01	0.22	F(5,64) = 4.91	< 0.01
Chlorophyll	0.0063	0.0035	1.77	0.08			
Zn	0.0083	0.0038	2.18	0.03			
P	− 0.0003	0.0002	− 2.01	0.048			
N	0.3660	0.2268	1.61	0.11			
**Beech** **Intra-specific**	**Coefficient**	**Std. Error**	**t**	**p-value**	**Adj. R**^**2**^	**F**	**p-value**
Elevation	− 0.0021	0.0007	− 2.97	< 0.01	0.09	F(2, 72) = 4.44	0.02
P	0.0003	0.0001	2.25	0.03

**Table 2:** Summary of variables contributing to intra-, and inter-specific bacterial community structure variation. Variation partition using PERMANOVA on Bray–Curtis distance on Hellinger-transformed reads. Testing was done “by margin”, using 999 permutations. For inter-specific bacterial community variation, pairwise PERMANOVA was also performed. Variable selection was performed using ordistep() prior to PERMANOVA analyses

Permanova					
	Variable	R^2^	F	DF	p-value
Intra-specific: spruce	Elevation	0.05	4.05	1	< 0.01
	Chlorophyll	0.04	3.38	1	< 0.01
	LMA	0.03	2.75	1	< 0.01
	Water content	0.03	2.72	1	< 0.01
	DBH	0.03	2.31	1	< 0.01
	Residual	0.81		64	
	Total	1.00		69	
Intra-specific: beech	Elevation	0.09	8.10	1	< 0.01
	P	0.04	3.53	1	< 0.01
	Ca	0.03	3.03	1	< 0.01
	N	0.03	2.71	1	< 0.01
	DBH	0.02	1.81	1	0.04
	Mg	0.02	1.94	1	0.02
	Residual	0.76		68	
	Total	1.00		74	
Inter-specific: Veluwe	Host species	0.48	11.94	3	< 0.01
Inter-specific: Bavaria	Host species	0.31	65.92	1	< 0.01

**Table 3:** Discriminant bacteria families for beech and spruce, based on ALDEX2 discriminant analyses

Biomarker	Phylum	Class	Order	Family	Effect size	Relative abundance beech	Relative abundance spruce
Beech	*Bacteroidota*	*Bacteroidia*	*Cytophagales*	*Hymenobacteraceae*	− 1.93	0.2701	0.0537
Beech	*Bacteroidota*	*Bacteroidia*	*Sphingobacteriales*	*Sphingobacteriaceae*	− 1.69	0.0760	0.0232
Beech	*Deinococcota*	*Deinococci*	*Deinococcales*	*Deinococcaceae*	− 1.36	0.0025	0.0005
Beech	*Proteobacteria*	*Alphaproteobacteria*	*Sphingomonadales*	*Sphingomonadaceae*	− 1.01	0.0626	0.0596
Beech	*Proteobacteria*	*Gammaproteobacteria*	*Burkholderiales*	*Oxalobacteraceae*	− 1.03	0.0243	0.0057
Spruce	*Armatimonadota*	*Armatimonadia*	*Armatimonadales*	*Armatimonadales*	1.08	0.0038	0.0291
Spruce	*Proteobacteria*	*Alphaproteobacteria*	*Caulobacterales*	*Caulobacteraceae*	1.60	0.0034	0.0315

### Within host-species differences

**Fig. 6:**
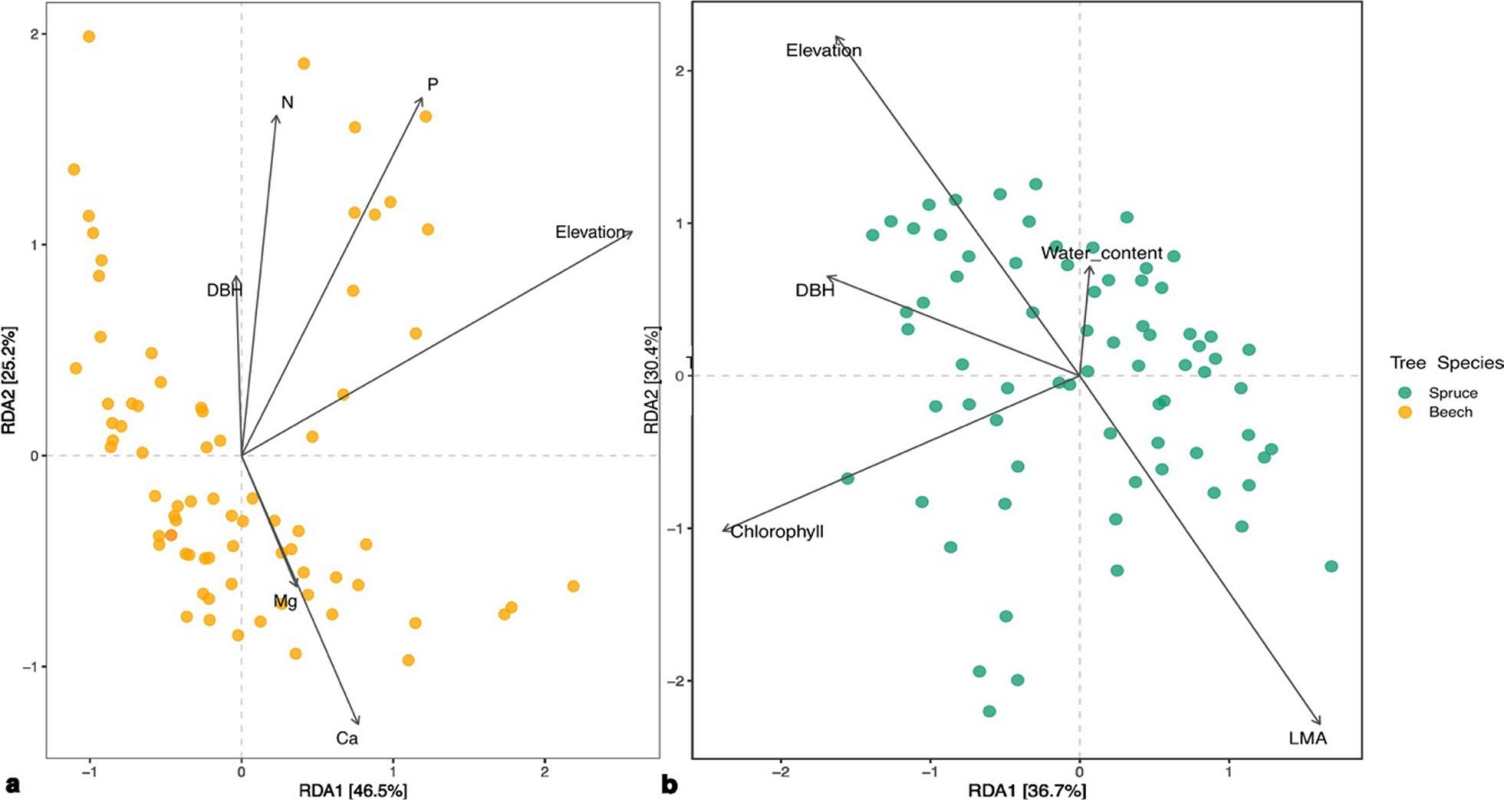
Bray–Curtis distance based RDA of beech (**a**) and spruce (**b**) phyllosphere bacterial community intra-host species variation and the significant (PERMANOVA: p < .05) variables explaining bacterial community composition. DBH: tree diameter at breast height, LMA: leaf mass per area

Considerable variations in bacterial alpha diversity was observed among individual trees of the same host species (Fig. 4, Additional file 1: Table S9). These intra-specific variations can be partially attributed to factors such as elevation and certain host traits (Table 1). We also observed significant intra-specific variations in the phyllosphere bacterial community compositions of beech and spruce (Fig. 6 and Table 2). These variations could not be explained by distance-decay (Additional file 1: Table S11) but can be attributed to elevation and certain host traits (Table 2). Twelve bacterial families in the spruce phyllosphere, were identified to be, either positively or negatively, associated with (p_BH_ < 0.05) elevation, needle chlorophyll, LMA, or needle water content (Table 4). Fifteen bacterial families were identified in the beech phyllosphere that, either positively and negatively, associated with (aldex2, p _BH_ < 0.05) elevation, leaf Ca or N (Table 4).

**Table 4:** Significant differentially abundant bacteria families in the spruce and beech phyllosphere identified by ALDEX2. (aldex.corr, and occurring in at least 30% of the samples)

Host Species	Biomarker	Spearman Rank correlation rho	Phylum	Class	Order	Family	Relative abundance
Spruce	Elevation	− 0.34	*Proteobacteria*	*Gammaproteobacteria*	*Diplorickettsiales*	*Diplorickettsiaceae*	0.0032
		− 0.40	*Proteobacteria*	*Alphaproteobacteria*	*Caedibacterales*	*Caedibacteraceae*	0.0026
		− 0.49	*Actinobacteriota*	*Actinobacteria*	*Corynebacteriales*	*Mycobacteriaceae*	0.0014
	Chlorophyll	0.37	*Actinobacteriota*	*Actinobacteria*	*Pseudonocardiales*	*Pseudonocardiaceae*	0.0024
		− 0.38	*Actinobacteriota*	*Actinobacteria*	*Corynebacteriales*	*Mycobacteriaceae*	0.0014
	Leaf mass per area	0.48	*Acidobacteriota*	*Acidobacteriae*	*Acidobacteriales*	*Acidobacteriaceae_(Subgroup_1)*	0.1728
		0.47	*Proteobacteria*	*Alphaproteobacteria*	*Acetobacterales*	*Acetobacteraceae*	0.1574
		0.38	*Actinobacteriota*	*Actinobacteria*	*Corynebacteriales*	*Mycobacteriaceae*	0.0014
		0.36	*Bdellovibrionota*	*Oligoflexia*	*0319-6G20*	*0319-6G20*	0.0020
		− 0.38	*Bacteroidota*	*Bacteroidia*	*Cytophagales*	*Spirosomaceae*	0.0165
		− 0.40	*Actinobacteriota*	*Actinobacteria*	*Frankiales*	*Nakamurellaceae*	0.0022
	Water content	0.37	*Actinobacteriota*	*Actinobacteria*	*Frankiales*	*Nakamurellaceae*	0.0022
Beech	Elevation	0.35	*Moraxellaceae*	*Proteobacteria*	*Gammaproteobacteria*	*Pseudomonadales*	0.0003
		0.31	*Burkholderiaceae*	*Proteobacteria*	*Gammaproteobacteria*	*Burkholderiales*	0.0021
		− 0.31	*Beijerinckiaceae*	*Proteobacteria*	*Alphaproteobacteria*	*Rhizobiales*	0.2884
		− 0.35	*Fimbriimonadaceae*	*Armatimonadota*	*Fimbriimonadia*	*Fimbriimonadales*	0.0003
		− 0.37	*AB1*	*Proteobacteria*	*Alphaproteobacteria*	*Rickettsiales*	0.0014
		− 0.42	*Bdellovibrionaceae*	*Bdellovibrionota*	*Bdellovibrionia*	*Bdellovibrionales*	0.0036
		− 0.44	*Chthoniobacteraceae*	*Verrucomicrobiota*	*Verrucomicrobiae*	*Chthoniobacterales*	0.0015
		− 0.47	*Diplorickettsiaceae*	*Proteobacteria*	*Gammaproteobacteria*	*Diplorickettsiales*	0.0004
		− 0.48	*Micavibrionaceae*	*Proteobacteria*	*Alphaproteobacteria*	*Micavibrionales*	0.0005
	Ca	0.38	*Kineosporiaceae*	*Actinobacteriota*	*Actinobacteria*	*Kineosporiales*	0.0014
		− 0.38	*Armatimonadales*	*Armatimonadota*	*Armatimonadia*	*Armatimonadales*	0.0038
		− 0.43	*Isosphaeraceae*	*Planctomycetota*	*Planctomycetes*	*Isosphaerales*	0.0020
	N	0.34	*Isosphaeraceae*	*Planctomycetota*	*Planctomycetes*	*Isosphaerales*	0.0020
		− 0.36	*Sphingobacteriaceae*	*Bacteroidota*	*Bacteroidia*	*Sphingobacteriales*	0.0770
		− 0.43	*Hymenobacteraceae*	*Bacteroidota*	*Bacteroidia*	*Cytophagales*	0.2573

## Discussion

The forest top canopy bacterial communities in the Veluwe area and Bavarian Forest National Park showed significant variation, both among host tree species (accounting for half of the variation in bacterial diversity and one-third of the variation in community composition) and within the same host tree species. Within the same tree species, elevation and host traits were identified as driving factors of subtle but significant variation in the phyllosphere bacterial community composition. We also identified 27 discriminant bacterial taxa associated with these driving factors. These bacterial markers could be further investigated as potential next-generation biomonitoring targets that can inform forest ecosystem change and alarm deterioration (e.g., due to soil acidification) [67].

### Within host species variation and its driving factors

Elevation was the most influential explanatory variable for the intra-specific variation in the phyllosphere bacterial communities of both beech and spruce in Bavarian National Park, potentially indicating the bacterial communities are sensitive to climatic conditions since elevation can be considered as a proxy for temperature [68]. For example, two predominantly predatory bacterial families *Bdellovibrionaceae* and *Micavibrionaceae*, were more abundant at lower elevations in the beech phyllosphere. However, this sensitivity was not equally observed for another group—*Myxococcaceae*, a facultative predatory bacterial family—which exhibited a consistent distribution across the elevational gradient. These divergent correlations could potentially be attributed to their different levels of flexibility in switching between obligate or facultative predatory mode and adjusting their prey range accordingly [69]. Two insects associated taxa (*Diplorickettsiaceae* and *Rickettsiales AB1*) also exhibited significant correlations with elevation, showing higher relative abundance at lower elevations in both beech and spruce phyllosphere. Given that lower elevations (especially in the case of the top canopy) tend to have a higher temperature, lower UV radiation, a richer insect fauna could be supported [70], potentially supporting elevated relative abundances of insect-associated bacterial families.

A suite of leaf traits also contributed to explaining the intra-specific variations in the phyllosphere bacterial communities. Specifically, leaf mass per area (LMA), a key metric in the leaf economics spectrum, along with leaf chlorophyll, leaf water content, and leaf N, P, Mg, Ca, Zn, correlated with the diversity, community structure and the relative abundance of various bacterial families in the community. The leaf economic spectrum is an important concept that defines the trade-off between plant resource acquisition and resource conservation, helping us understand plant adaptation to different environments [66]. This study shows that, within the same host species, this trade-off also influences hosts’ interaction with their microbial communities. We observed, for example, an association between the plant-beneficial bacteria family *Pseudonocardiaceae* and higher spruce needle chlorophyll. Members of the *Pseudonocardiaceae* family have known plant-beneficial traits including anti-bacterial, anti-fungal, and anti-tumor [71, 72], potentially explaining their heightened abundance in healthier spruce trees, inferred from their high chlorophyll content [73]. We also observed bacterial families that are crucial for plant nitrogen and phosphorus utilization (e.g., *Isosphaeraceae*, [74, 75]) to be associated with higher beech foliar N, pointing to phyllosphere bacteria’s potential role in leaf nutrients fixation or utilization. With more readily available nitrogen (i.e., through aerial deposition), these bacterial families could lose their competitive advantage over other plant-associated microbes, including pathogens [76], resulting in shifts in plant host functioning. While 24% and 19% of the within host species variation was explained in our study for beech and spruce, respectively, the remaining variance remained unexplained. This is unsurprising, considering the significant variation that exists within individual hosts [2, 77].

We observed variation in bacterial diversity along beech and spruce’s foliar Mg and P concentration gradients, two variables that have also been linked to soil acidification and nitrogen deposition [78, 79]. A lower needle Mg, among other essential alkaline macro-nutrients, can be a sign of nutrients leaching in soil from acid rain and aerial nitrogen deposition [79]. The higher cuticular permeability in Mg-deficient spruce needles [80], which makes the needle substrates more readily available, likely promote higher bacterial diversity. We also found lower leaf P to be associated with lower beech phyllosphere bacterial diversity. Lower beech foliar P has been shown to be one of the many ramifications of soil nitrification and acidification during the last two decades [78]. These potential chain reactions point to the complexity of ecosystem functioning, and how changes in soil pH could have rippling effect up towards to the top canopy residing bacteria.

### Host species effect and core microbiome

Our results demonstrated a relatively strong host species effect (R^2^ = 0.33, p < 0.01). Notably, tree species that were more phenotypically dissimilar (deciduous versus coniferous) also harbored more distinct phyllosphere bacterial communities, indicating possible phylosymbiosis—a pattern in which the similarity of host-associated microbiome reflects the evolutionary relatedness of the host [81]. In addition to clustering patterns based on host tree species, we identified bacterial taxa discriminately abundant in either beech or spruce. The known characteristics of taxa discriminately abundant in either beech or spruce align well with the different leaf physiologies between needles and broadleaves: beech leaves, thinner and with larger leaf surface, were characterized by multiple common plants epiphytic [82–84] and UV-tolerant [85] bacterial groups (i.e., *Sphingobacteriaceae*, *Oxalobacteraceae*, and *Deinococcaceae*); while long, cylindrical spruce needles, with more leaf mass per leaf area, were characterized by *Caulabacteraceae*, a primarily endophytic [86] and lignin-degrading [87] bacterial family*.* Within phenotypically similar hosts, bacterial diversity showed the greatest difference between spruce (mean and se: 4.76 ± 0.06) and Scots pine (mean and se: 2.31 ± 0.32). This aligns with Scots pine being a pioneer species adapted to dryer and harsher environments compared to spruce, leading to a lower microbial diversity [88–91]. These observations also constituted as baseline knowledge on Scots pine (*Pinus sylvestris)*, silver birch (*Betula pendula)*, and silver fir (*Abies alba)*, for which there is very limited knowledge available (but see: [92]).

Despite a pronounced host species effect, we also detected a core microbiome of four bacterial taxa that were invariably present in all six tree species. This finding is in line with several studies highlighting the existence of a core microbiome in the phyllosphere of both woody and non-woody plants [2, 18, 93, 94]. Their consistent presence across different host trees suggests that these four taxa, belonging to the *Beijerinckiaceae* and *Acetobacteraceae* family, play important ecological roles within the phyllosphere. This is exemplified by the versatile *Beijerinckiaceae* family, known for their ability to carry out methanotrophy, methylotrophy and nitrogen fixation in acidic soil environments [95, 96]. One of the core taxa within this family belongs to the genus *1174-901-12*, the most abundant genus in beech and spruce. Besides in the phyllosphere, *1174-901-12* has also been found on abiotic surfaces such as roof tiles [97] and photovoltaic panels [98]. This multitude of metabolic capabilities and ecological flexibility may explain members of this family being successful generalists in the temperate forest, utilizing both deciduous and coniferous leaf substrates. Although one might also expect coniferous species to have a higher number and abundance of core taxa compared to deciduous hosts due to longer, multi-year lifespan of coniferous needles, the opposite was observed between beech and spruce (Additional file 1: Table S7).

## Conclusions

Our extensive survey of the forest top canopy bacterial communities highlighted the importance of host species identity and the substantial variation within a host tree species. Investigating these intra-specific variations can help us understand how trees and their microbiomes adapt together to their environment. At the individual tree level, bacterial communities varied with both host tree traits and climatic conditions. Scaling up to the forest landscape, the measurable differences in the phyllosphere bacterial communities with regards to highly plastic leaf traits demonstrated that the top canopy phyllosphere microbiome is sensitive to changes in forest conditions. Just as keystone species (i.e., species of special importance in maintaining the ecological communities [99]) can serve as early indicators of ecosystem health, monitoring key bacterial groups (e.g., identified discriminant and core taxa) in the phyllosphere could provide valuable insights into host trees adaptation and, at scale, the functioning of forest ecosystems. Incorporating these insights into eDNA monitoring strategies holds the potential to enhance our ability to detect and respond to changes in forest ecosystems [100], particularly in the context of ongoing environmental challenges such as drought, pest outbreaks, and aerial nitrogen deposition. [101]. Building on these insights, we recommend the phyllosphere microbiome to be considered as a candidate for forest monitoring. Among the multiple layers of forest canopy (e.g., the bottom and middle layers), the top canopy may of particular interest, given large scale monitoring efforts often rely on remote sensing of the sunlit upper canopy [102]. Beyond being an indispensable part of biodiversity, forest phyllosphere microbiomes hold the potential to manifest forest ecosystem change, and provide early warning signs.

### Supplementary Information

**Additional file 1:** Contains supporting tables and figure.

**Additional file 2:** Contains processed DNA sequencing results and metadata.

## Data Availability

All data suporting the findings of this study are available within the paper and its Additional files 1 and 2.
