# Supplementary material for: Forest top canopy bacterial communities are influenced by elevation and host tree traits
Source: Environ Microbiome. 2024 Apr 5;19:21. doi: 10.1186/s40793-024-00565-6 (PMC10998314; doi:10.1186/s40793-024-00565-6)
Supplement: Supplementary file 1 — Additional file 1: Contains supporting tables and figure. [file 40793_2024_565_MOESM1_ESM.docx]

Forest top canopy bacterial communities are influenced by elevation and host tree traits

Yiwei Duan^1*^, Andjin Siegenthaler^1^, Andrew K. Skidmore^1^, Anthony A. Chariton^2^, Ivo Laros^3^, Mélody Rousseau^1^, G. Arjen de Groot^3^

# Supplementary material 1

Supplementary Table S1. Difference in bacterial communities between different field preservation methods - frozen vs. snap frozen. n = 10, paired.

|  | Plant reads % | Alpha diversity -Shannon | Community composition |
| --- | --- | --- | --- |
| Snap-frozen vs. Frozen | p = 0.28 | p = 0.23 | p = 0.955 |
| Plant reads % and alpha diversity were tested using paired Wilcoxon signed rank exact test | | | |
| Potential community composition distortion was tested using PERMANOVA (adonis2) | | | |

Supplementary Table S2. PCR reagents and spike-ins for bacterial 16S rRNA gene amplifications of leaf DNA extracts

| **Component** | | **Supplier** | | **Final Concentration** | **Volume (µl)** |
| --- | --- | --- | --- | --- | --- |
| H_2_O | | Sigma | | NA | 2.525 |
| PCR buffer | | Thermofisher | | 1x | 1.25 |
| MgCl_2_ | | Thermofisher | | 2.5 mM | 0.625 |
| Trehalose | | Thermofisher | | 4% | 5.0 |
| Bovine serum albumin | | VWR | | 200 ng/µl | 0.125 |
| dNTP | | VWR | | 200 µM | 0.25 |
| PrimerMix^1^ | | Biolegio | | 250 µM | 0.125 |
| mPNA^2^ | | PNA Bio Inc. | | 1 µM | 0.25 |
| pPNA^3^ | | PNA Bio Inc. | | 1 µM | 0.25 |
| Platinum Taq Polymerase | | Thermofisher | | 0.08 U/µl | 0.10 |
| Subtotal | |  | |  | 10.5 |
| DNA template | | N/A | | N/A | 2.0 |
| ^1^ Primer sequence  (5' to 3')* | 515F_CS1: ACACTGACGACATGGTTCTACAGTGYCAGCMGCCGCGGTAA  806R_CS2: TACGGTAGCAGAGACTTGGTCTGGACTACNVGGGTWTCTAAT | | | | |
| ^2^mPNA sequence (mitochondria PNA)  ^3^pPNA sequence (plastid PNA) | | | ggcaagtgttcttcgga  ggctcaaccctggacag | | |
| * The primer adapter sequences (CS1/CS2) are underlined. | | | | | |
| Synthetic spike-in sequence (5' to 3')  Spike-in:  CTTGTACGCCGAGGCCCGACGCGCCAGGAGTACTGCTTTGATCCAGAAAGTAGGTCATCCATCGCAACGGTCAATGACCGTTGGTAGAAGTCTGGCCCAAGGTCCCGGCTGCGCTGGGACGGTAGTGGCGACTCGTTAAATGGTCTTCGCTTGATGATTAGTCTTGTCAGCCGCTATCCTACTTTGCTTACTGGTCAGTCATGCTAAACGCGGTCCTTACGAGGGCGCTTAGTGTTTCTTGCAAAGCGGGTTGCTAGCCTACCTGAATAGAGACGCAGTGTGAAGCGTCCT  Note: The sequence was randomly generated *in silico* using R. The synthetic sequence was the same length as the amplified products (~300 bp), and contained no more than three homologous nucleotides. Prior to synthesis, primers were added to the synthetic sequence with the synthetic sequence synthesized by Integrated DNA Technologies, Inc | | | | | |

Supplementary Table S3. PCR program for bacterial 16S rRNA gene amplification of DNA extracts

| **Step** | **Temperature** | **Time** |
| --- | --- | --- |
| 1 | 94°C | 2 min |
| 2 | 94°C | 30 sec |
| 3 | 75°C | 10 sec |
| 4 | 56°C | 3 min  -1°C/cycle |
| 5 | 72°C | 1 min |
| 6 | Go to Step 2, 14x | |
| 7 | 94°C | 30 sec |
| 8 | 75°C | 10 sec |
| 9 | 42°C | 3 min |
| 10 | 72°C | 1 min |
| 11 | Go to Step 7, 19x | |
| 12 | 72°C | 10 min |
| 13 | 10°C | For ever |
| 14 | End |  |

Supplementary Table S4. Bioinformatic pipelines for bacterial 16S rRNA gene metabarcoding data. Steps 2-6 were conducted in QIIME 2™ and steps 7-13 in R version 4.2.3. Non-default parameters are provided.

| **Step** | **Package** | **Parameters** | **Reads** | **ASVs** | **Samples** |
| --- | --- | --- | --- | --- | --- |
| 1. Demultiplexing |  |  | 173,874,448 (per sequencing direction) |  | 493 |
| 2. Primer trimming | cutadapt | minimum-length: 200 | 168,445,114 (per sequencing direction) |  | 493 |
| 3. Quality filtering | dada2 | trunc-len-f: 232  trunc-len-r: 230 | 147,625,330 |  | 493 |
| 4. Denoising | dada2 | MaxEE: 2 | 143,571,800 |  | 493 |
| 5. Paired reads merging | dada2 |  | 130,817,930 |  | 493 |
| 6. Chimera removal) | dada2 |  | 116,120,466 | 149,143 | 493 |
| 7. Post-clustering curation | LULU | minimum_match: 90%^1^  minimum_relative_cooccurence: 0.95 | 116,120,466 | 113,598 | 493 |
| 8. Blank correction | Custom R-script | Removal criteria: max reads in blanks ≥ max reads in samples | 112,363,185 | 112,923 | 478* |
| 9. Non-bacterial reads filter | Custom R-script | Removal of reads with domain ≠ bacteria; order = chloroplast; and family = mitochondria | 79,405,492 | 104,499 | 478 |
| 10. Tag-switching | Custom R-script | Tag-switching threshold: 0.003% | 79,400,076 | 104,499 | 478 |
| 11. Sample selection | Custom R-script | Exclusion of samples that are not pertinent to this study | 17,857,041 | 13,496 | 239 |
| 12. Low frequency noise filtering | Custom R-script | Removal of ASVs with < 5 reads in total | 17,847,008 | 9936 | 239 |
| 13. Sample selection and Rarefaction | Custom R-script and Vegan | Exclusion of samples used for methodological control (PNA clamps and freezing methods)  Rarefaction depth: 10312 reads | 2,175,905 | 8972 | 211 |

* Removal of 6 positive control samples and 9 negative control samples.

1. Brandt, M. I. *et al.* Bioinformatic pipelines combining denoising and clustering tools allow for more comprehensive prokaryotic and eukaryotic metabarcoding. *Mol Ecol Resour* **21**, 1904-1921, doi:10.1111/1755-0998.13398 (2021).

Supplementary Table S5. Plant reads per species, test results using 1uM or no PNA clamps.

| **Tree Species** | **Plant reads percentage (mean ± sd)** | **Test: PNA-free plant reads percentage**  **(mean ± sd)** | **Test: 1uM-PNA plant reads percentage**  **(mean ± std)** |
| --- | --- | --- | --- |
| Oak | 75.2 ± 30.6 | 98.8 ± 1.6 | 75.3 ± 34.8 |
| Beech | 61.8 ± 20.4 | 95.3 ± 3.4 | 74.9 ± 19.9 |
| Pine | 80.8 ± 11.7 | 92.6 ± 2.2 | 71.7 ± 6.4 |
| Spruce | 58.0 ± 19.9 | 76.8 ± 21.2 | 60.7 ± 24.0 |
| Fir | 39.9 ± 22.0 | - | - |
| Birch | 83.2 ± 8.4 | - | - |


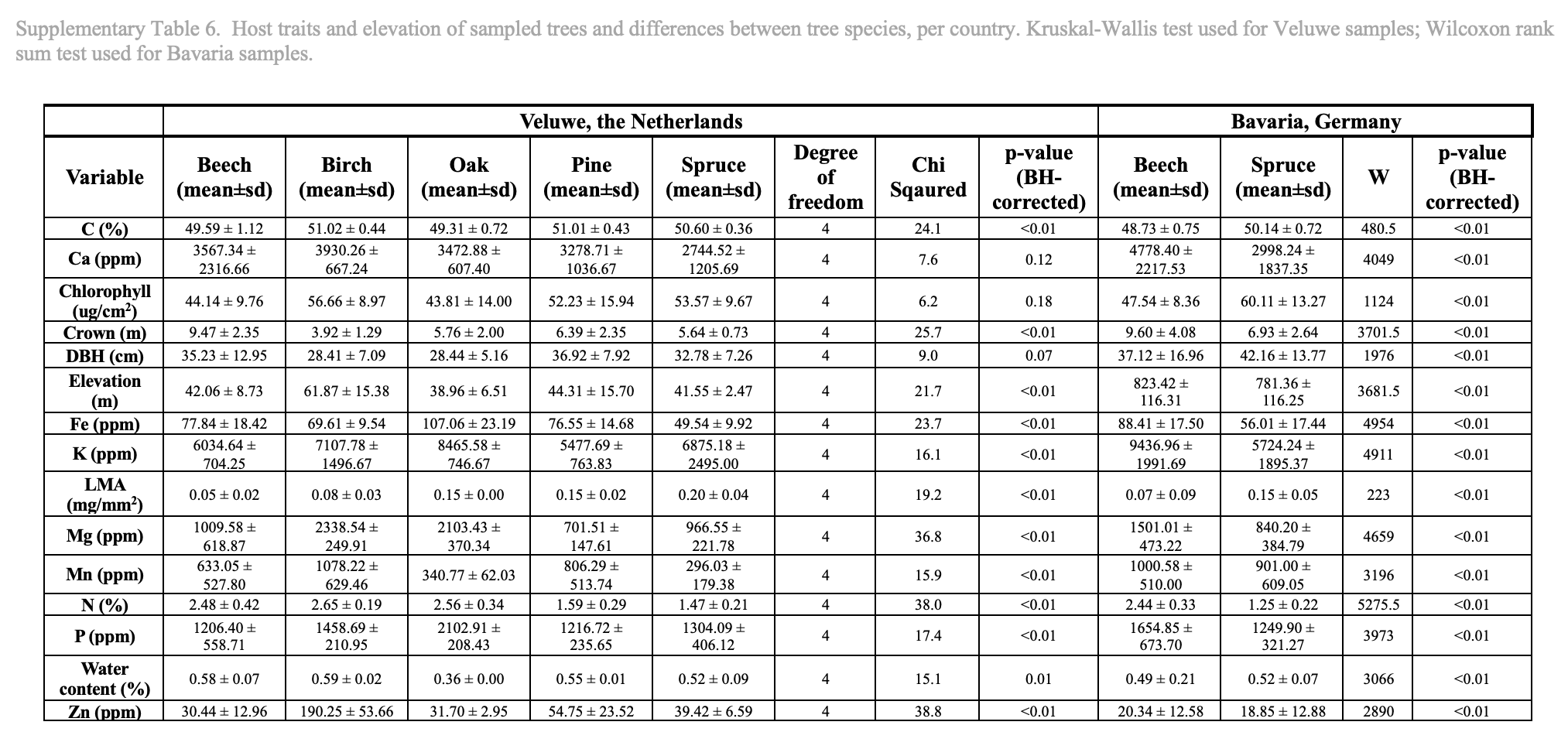


Supplementary Table S7. Core taxa of and their relative abundance. Defined as present in >95% of the samples.

| **Host** | **Phylum** | **Class** | **Order** | **Family** | **Genus** | **Taxa reads to total reads ratio** | **Core taxa reads to total reads ratio** |
| --- | --- | --- | --- | --- | --- | --- | --- |
| All samples | Proteobacteria | Alphaproteobacteria | Rhizobiales | Beijerinckiaceae | 1174−901−12 | 0.08 | 0.17 |
|  |  |  |  |  | NA | 0.04 |  |
|  |  |  | Acetobacterales | Acetobacteraceae | Acidiphilium | 0.02 |  |
|  |  |  |  |  | NA | 0.02 |  |
| Spruce | Proteobacteria | Alphaproteobacteria | Rhizobiales | Beijerinckiaceae | 1174−901−12 | 0.05 | 0.23 |
|  |  |  |  |  |  | 0.04 |  |
|  |  |  |  |  | NA | 0.07 |  |
|  |  |  | Acetobacterales | Acetobacteraceae | Acidiphilium | 0.02 |  |
|  |  |  |  |  |  | 0.03 |  |
|  | Acidobacteriota | Acidobacteriae | Acidobacteriales | Acidobacteriaceae  (Subgroup_1) | Terriglobus | 0.02 |  |
| Beech | Proteobacteria | Alphaproteobacteria | Rhizobiales | Beijerinckiaceae | 1174−901−12 | 0.09 | 0.17 |
|  |  |  |  |  | NA | 0.02 |  |
|  |  |  | Acetobacterales | Acetobacteraceae | Acidiphilium | 0.03 |  |
|  |  |  |  |  | NA | 0.03 |  |

Supplementary Table S8. Alpha Diversity (Shannon index and ASV Richness) Pairwise Wilcoxon rank sum test with continuity correction between beech, spruce, birch, oak, and pine from the Veluwe National Park (NL), and beech and spruce from Bavarian Forest National Park (DE).

P value adjustment method: Benjamini-Hochberg

|  |  |  | Shannon |  |  |  |  | ASV Richness |  |  |
| --- | --- | --- | --- | --- | --- | --- | --- | --- | --- | --- |
|  |  | Beech | Birch | Oak | Pine |  | Beech | Birch | Oak | Pine |
|  | Birch | 0.62 | - | - | - | Birch | 0.28 | - | - | - |
| Veluwe | Oak | 0.43 | 0.43 | - | - | Oak | 0.63 | 0.98 | - | - |
|  | Pine | 0.01 | <0.01 | 0.09 | - | Pine | 0.14 | <0.01 | 0.06 | - |
|  | Spruce | 0.84 | 0.96 | 0.42 | <0.01 | Spruce | 0.75 | 0.48 | 0.67 | 0.06 |
| Bavaria | W= 460, p < 0.01 | | | | | W= 697.5, p < 0.01 | | | | |

Supplementary Table S9. Alpha Diversity of tree species in The Veluwe National Park, the Netherlands and Bavarian Forest National Park, Germany.

|  |  | Veluwe | | | | | | | |
| --- | --- | --- | --- | --- | --- | --- | --- | --- | --- |
|  |  | Beech | Spruce | | Pine | Birch | | Oak | Fir |
| Shannon | Mean ± se | 4.07 ± 0.23 | 4.03 ± 0.21 | | 2.31 ± 0.32 | 3.90 ± 0.12 | | 3.46 ± 0.32 | 4.33 ± 0.13 |
|  | Range | (3.17, 4.94) | (3.00, 4.95) | | (0.60, 4.30) | (2.30, 4.90) | | (2.27, 4.39) | (4.21, 4.46) |
| ASV Richness | Mean ± se | 235 ± 33 | 255 ± 22 | | 157 ± 22 | 289 ± 19 | | 280 ± 33 | 269 ± 116 |
|  | Range | (114, 331) | (141, 347) | | (49, 288) | (136, 481) | | (165, 393) | (153, 384) |
|  |  | Bavaria | | | | | | | |
|  |  | Beech | | Spruce | | | Fir | | |
| Shannon | Mean ± se | 3.93 ± 0.06 | | 4.85 ± 0.05 | | | 4.64 ± 0.11 | | |
|  | Range | (1.75, 5.05) | | (3.57, 5.70) | | | (4.47, 4.96) | | |
| ASV Richness | Mean ± se | 302 ± 13 | | 523 ± 18 | | | 543 ± 35 | | |
|  | Range | (72, 661) | | (213, 869) | | | (501, 646) | | |

Supplementary Table S10. Bacterial community composition (Bray-Curtis)) Pairwise comparison between beech, spruce, birch, oak, and pine from Veluwe National Park using PERMANOVA, P value adjustment method: Holms.

| Pairwise PERMANOVA | | | | |
| --- | --- | --- | --- | --- |
| **Pairs** | **R^2^** | **F** | **DF** | **Pr(>F)** |
| Pine vs Oak | 0.38 | 11.69 | 1 | < 0.01 |
| Pine vs Beech | 0.47 | 16.80 | 1 | < 0.01 |
| Pine vs Spruce | 0.41 | 14.68 | 1 | < 0.01 |
| Pine vs Birch | 0.46 | 26.65 | 1 | < 0.01 |
| Oak vs Beech | 0.19 | 2.81 | 1 | < 0.01 |
| Oak vs Spruce  Oak vs Birch  Beech vs Spruce  Beech vs Birch  Spruce vs Birch | 0.29  0.22  0.37  0.29  0.32 | 5.82  6.77  8.11  9.60  12.07 | 1  1  1  1  1 | < 0.01  < 0.01  < 0.01  < 0.01  < 0.01 |

Supplementary Table S11. Distance-decay analysis of beech and spruce bacterial communities using Mantel test with Pearson correlation (999 permutations).

| **Mantel test** | | |
| --- | --- | --- |
| **Tree specie** | **r** | **p** |
| Beech | - 0.02 | 0.77 |
| Spruce | 0.06 | 0.06 |


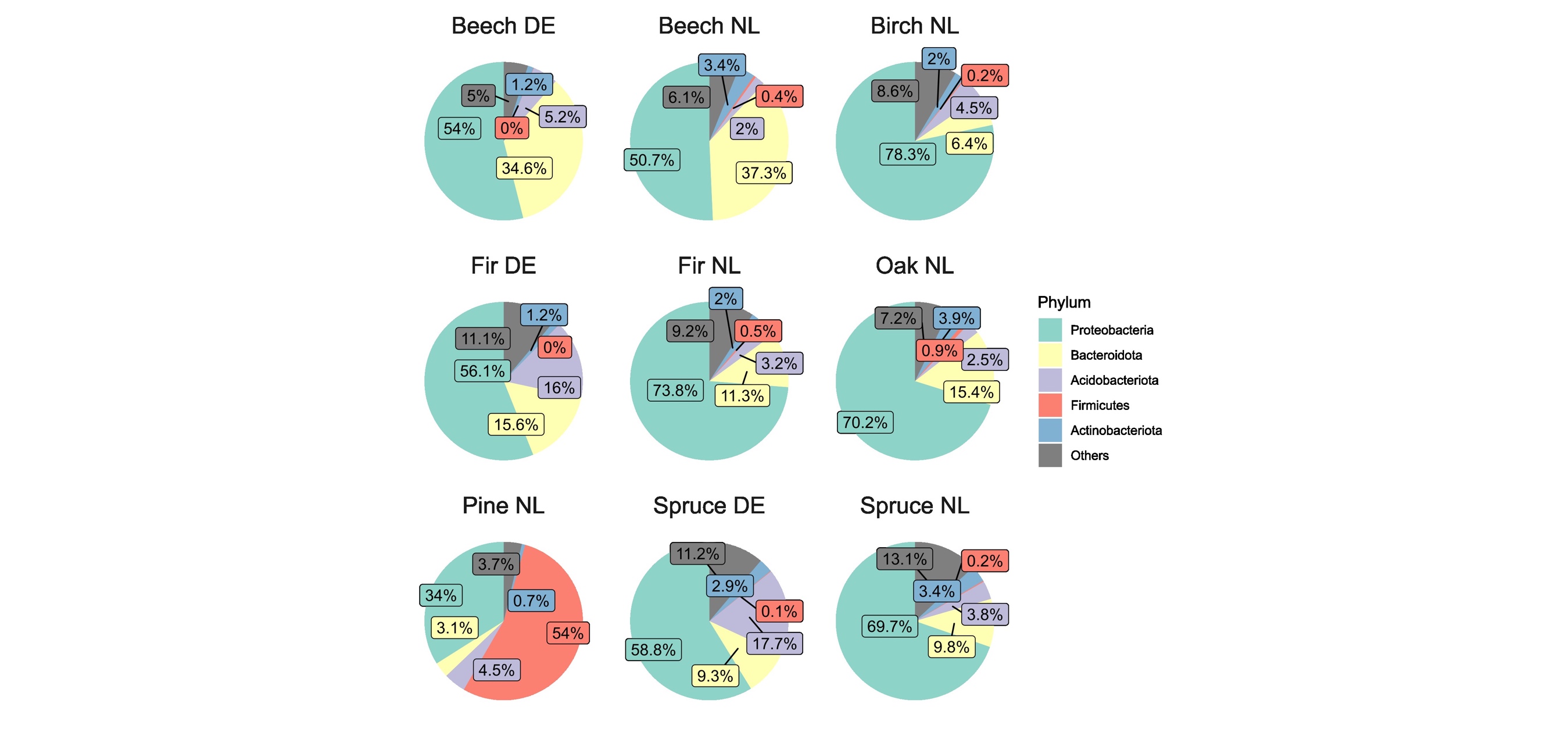


Supplementary Figure S1. Top five phyla in the bacterial communities of temperate forest phyllosphere, shown per country. Percentages represent relative read abundance. Samples collected in Bavarian National Park, Germany (DE) and The Veluwe National Park, the Netherlands (NL).

Supplementary Figure S2. ASV Accumulation curves of samples collected in two study forests from six study tree species. Fagus sylvatica (European beech), Picea abies (Norway spruce), Quercus robur (European oak), Pinus sylvestris (Scots pine), Abies alba (silver fir), and Betula pendula (European birch).
